# Woman‐centeredness of family planning care and associated factors in a semi‐urban health district in West Cameroon

**DOI:** 10.1002/ijgo.70654

**Published:** 2025-11-17

**Authors:** Jovanny Tsuala Fouogue, Wiliams Carter Kenne Djuatio, Armand Tiotsia Tsapi, Ymele Florent Fouelifack, Madye Ange Ngo Dingom, Estelle Diane Modjo Kamdem, Jeanne Hortence Fouelifack Fouedjio

**Affiliations:** ^1^ Department of Infectious Disease Epidemiology and International Health London School of Hygiene and Tropical Medicine London UK; ^2^ West Regional Delegation for Public Health Ministry of Health Bafoussam Cameroon; ^3^ Faculty of Sciences and Technologies Evangelical University Institute of Cameroon Mbouo Bandjoun Cameroon; ^4^ Department of Surgery and Specialities Higher Institute of Medical Technologies Yaounde Cameroon; ^5^ Department of Gynecology, Obstetrics and Maternal Health Faculty of Medicine and Pharmaceutical Sciences of the University of Dschang Dschang Cameroon

**Keywords:** compassionate care, contraception, correlates, determinants, family planning, patient‐centered, quality of care, woman‐centeredness

## Abstract

**Objective:**

To measure the woman‐centeredness of family planning (FP) care and determine its correlates in West Cameroon.

**Methods:**

We conducted a cross‐sectional analytical study from August to November 2024 in the Mifi Health District (MHD). We included women receiving FP care in all the public health facilities. We collected data were using the person‐centered FP care scale (PCFPS). Descriptive and inferential statistics were computed with R software. Respondents' characteristics were summarized, and woman‐centeredness scores computed using the PCFPS guide. We used the cutoff threshold technique to distinguish high and low scores. Bivariate and multivariate linear regressions were conducted to determine the correlates of woman‐centeredness of FP care. Regression coefficients with their 95% confidence intervals (CIs) were computed with a significance threshold of 5%.

**Results:**

The median (range) woman‐centeredness score for the 179 respondents was 73.33% (12.22–88.88). Specifically, the median (range) score (76.38% [8.33–91.66]) for woman's respect and autonomy was higher than that (61.11% [5.55–100]) for health facility environment. The FP care woman‐centeredness score at the district hospital was seven‐point higher than in first‐level primary healthcare facilities (a.Coefficient: 7.33; 95% CI: 1.11–13.56; *P* = 0.02). Likewise the woman‐centeredness score of FP care for women with a monthly income ≤100 USD was significantly lower than for women earning >300 USD monthly (a.Coefficient: –6.98; 95% CI: −12.96 to −1.01; *P* = 0.02).

**Conclusion:**

FP care in the MHD was highly women centered. However, FP care was less likely to be woman‐centered for low‐income women and for those attending first‐level primary health care facilities.

## 1 INTRODUCTION

Family planning (FP) is defined by the WHO as “the ability of individuals and couples to anticipate and attain their desired number of children and the spacing and timing of their births. It is achieved through use of contraceptive methods and treatment of involuntary infertility.”^1^ Optimal uptake of FP services yields a wide range of health and non‐health dividends including reduction of maternal and infant morbidity and mortality, expanded education and empowerment opportunities for women, sustainable demographic growth, enhanced economic prosperity, and mitigation of the burden of climate change.^2^, ^3^ Mindful of that, the global health community has adopted FP as a sustainable development goal (SDG) setting a desirable level of 75% women of reproductive age (15–49 years) having their needs for family planning satisfied with modern contraceptive methods by 2030.^4^ Yet, low‐ and lower‐middle income countries that badly need to harness the potential of adequate coverage with responsive FP services in view of tapping into those dividends are not on track to reaching the related sustainable development goal desired outcome.^4^ For instance, in sub‐Saharan Africa (SSA), little progress has been made on meeting FP needs with modern contraceptive methods as the coverage rose from 52% in 2015 to 58% in 2022 hence remaining a moderate achievement.^4^, ^5^ Commitment by global public health bodies to increase access to and uptake of modern contraceptive methods is challenged by numerous barriers whose magnitude varies between and within countries.^6^ These include limited choices of contraceptive methods; limited access to FP services; fear or experience of side‐effects of FP methods; cultural or religious opposition to FP; poor quality of available FP services; users' and providers' bias against some FP methods; and gender‐based barriers to accessing FP services.^2^ Among the previous, the poor quality of FP services deserves special attention for two reasons: first, expanding access to FP should logically begin with improvements of already available services; second, the ongoing implementation of the global agenda on universal health coverage is subject to the provision of quality health services.^7^, ^8^, ^9^

Cameroon is a SSA lower‐middle income country with a total fertility rate estimated at 4.8 in 2018, a quite high maternal mortality ratio (258 maternal deaths for 100 000 live births), a moderate human development index (0.588 in 2024), and a considerable gender inequality index (0.558 in 2023).^10^, ^11^, ^12^, ^13^ Despite a continuous rise since the 1990s, the FP need met by modern contraceptive methods stood at a modest 36% in 2018 and is projected to be less than 40% by 2025.^5^, ^11^ Meanwhile, the proportion of unmet FP needs stalled at 25%.^5^ In implementing its nationwide public health strategic plan for 2019–2027 the country launched several national family planning 2030 commitments centered on well‐thought priorities including: raising contraceptive prevalence from 15% to 35% and lowering unmet FP need from 23% to 10% alongside quality improvements in service delivery.^14^, ^15^ In the same vein, the United Nations Population Funds (UNFPA) Regional Office for West and Central Africa, in line with global commitments towards better people's health outcomes, endeavored to support Cameroon in delivering quality FP services among other strategic priorities.^16^ Indeed, historically, the global health movement to improve the quality of FP services adopted successive technical frameworks all having at their core women experience, woman‐centeredness and women satisfaction.^17^, ^18^, ^19^, ^20^

Critical to achieving the quality improvements underpinning higher FP take up is understanding current shortcomings of FP services countrywide. To date, the handful of available studies on the topic underscores significant associations between the quality of services and uptake of FP methods but no published research has explored the women‐centeredness of FP care.^21^, ^22^, ^23^ Nevertheless, evidence from other SSA countries through quality assessment indicates that FP services are not sufficiently centered on womens' needs and preferences.^1^, ^24^, ^25^, ^26^, ^27^

The present study aimed to measure the women‐centeredness of FP services and to determine its correlates in a semi‐urban health district in the West Region of Cameroon.

## 2 MATERIALS AND METHODS

### 2.1 Study design, period and setting

#### Design and period

We conducted a cross‐sectional analytical study in the Mifi Health District (MHD) from August 19, 2024, to November 21, 2024.

#### Setting

The MHD in one of the 20 districts of the West Region of Cameroon and is home to the Region's capital city. The population of the MHD was estimated at 455,974 inhabitants in 2024 and the total fertility rate in the West Region was 5.5 while the country rate stood at 4.8 in 2018.^11^, ^28^ The met need for family planning in the Region (18%) is close to the country average (19%). In the West Region, 96.9% of childbirths are facility‐based and the maternal mortality ratio stood at 467 deaths/100 000 live births in 2018.^11^

The MHD is made up of rural and urban areas and comprises 28 health facilities run by the Ministry of Health. These include 22 first‐level primary health facilities (called “integrated health centers”), three second‐level primary health facilities (“called subdivisional medical centers”), one first‐level referral hospital (called “Mifi District Hospital [MDH]”) and one second‐level referral hospital (called “Bafoussam Regional Hospital [BRH]”).

Modern family planning methods are delivered at highly subsidized prices in all the public health facilities of the MHD and across the country. The following FP methods are routinely available: progestin‐only oral contraceptives, combined oral contraceptives, intramuscular progestins and subcutaneous progestins implants. The District Health Information System‐2 indicates that 21 060 women adopted a modern contraceptive method in the MHD in 2022.^11^

### 2.2 Study population

#### 2.2.1 Sample size

The minimum sample size was calculated using the following formula $n=z_{1-\frac{\alpha }{2}}^{2}P\left(1-P\right)/d^{2}$ where *n* is the minimal sample size; *P* is the anticipated proportion of adequate woman‐centeredness of family planning services (set at 30%); *d* is the absolute precision on either side of the proportion (set at 7%); *Z* is the 95% *Z*‐score and *α* the significance level (set at 0.05).^29^ The calculated crude minimal sample size was 165. After adding a 5% margin of error the final minimal sample size was 173.3, rounded up to 174 respondents.

#### 2.2.2 Sampling

##### 2.2.2.1 Inclusion criteria

We followed a proportional stratified non‐probability sampling approach to select health facilities then women. We deemed that approach best suited to account for the experience of FP care at each health facility in the whole sample.

##### 2.2.2.2 Selection of health facilities

Using the DHIS‐2 we considered all the health facilities that recorded at least 10 new FP users in 2023. We further calculated the percentages of individual health facilities in the MHD total number of new FP users recorded in 2023. The sample size was distributed to those facilities proportionally to the previous percentages.

##### 2.2.2.3 Selection of participants

From the records of the FP unit of each selected health facility, we retrieved the names of women aged ≥21 years whose telephone numbers were available. They were consecutively invited by telephone beginning with the most recent visit and moving backward until the number of participants needed for the index health facility was obtained. Face‐to‐face appointments were arranged, and women signed the informed consent form before answering survey questions that focused on their most recent FP visit.

##### 2.2.2.4 Exclusion criteria

Women who withdrew their consent during the survey and those who did not answer all the survey questions were excluded.

### 2.3 Ethics

The protocol of this research study was approved by the Regional Ethics Committee for Human Research (Reference N° 770/31/07/2024/CE/CERSH‐OU/VP). The study procedures complied with the best practices of research involving human subjects set by the 2013 version of Helsinki declaration and all relevant Cameroonian regulations. Women were receive no compensation for their participation in the study.

### 2.4 Data collection

Enumerators conducted face‐to‐face interviews at to women's preferred locations to prevent them from from traveling. They completed the survey form deployed on electronic tablets through an open data kit (ODK Inc., San Diego, California) form. At the end of each survey, answers were recorded and saved on a central server. The questionnaire used in this study was adapted, translated, and back translated from the person‐centered family planning (PCFP) care scale developed and validated in India and Kenya.^30^ It was then pretested on 10 female FP users from a neighboring health district whose answers were not included in the final dataset.

### 2.5 Data management and statistical analyses

The final dataset was cleaned, coded and extracted from the open data kit (ODK) server. Descriptive statistics were computed using R software version 2.4.1 via its interface R‐cmdr (GPL2/GPL3). Qualitative variables were summarized as proportions and quantitative variables were summarized using position parameters (means with standard deviations [SD] and medians with interquartile ranges [IQR] depending on their distribution). Woman‐centeredness scores were computed based on the PCFP scale guide for individual respondents, for individual health facilities and for each level of health facilities.^30^ Answers to survey questions were assigned pre‐defined numerical integer values from 0 to 4 and the scores were calculated by adding up those values. The cutoff threshold technique was used to distribute participants according to their women‐centeredness scores in two categories (high and low scores). The cutoff value was computed with the following formula^31^, ^32^:

$$\mathrm{Cutoff}\;\text{values}=\left(\frac{\text{Highest score}-\text{Lowest score}}{2}\right)+\text{Lowest score}$$

Inferential statistics were based on linear regression analyses. The dependent variable was the woman‐centeredness score, and independent variables included respondents' sociodemographic characteristics (occupation, level of education, marital status, income category, and parity) and health system features (category of health facility, gender of the FP provider, and the FP method). Bivariate analyses were first carried out, followed by a multivariate model. Regression coefficients with their 95% confidence intervals (CIs) were computed alongside their *P* values with a threshold for significance set at 5%.

## 3 RESULTS

### 3.1 Sample characteristics

**FIGURE 1:**
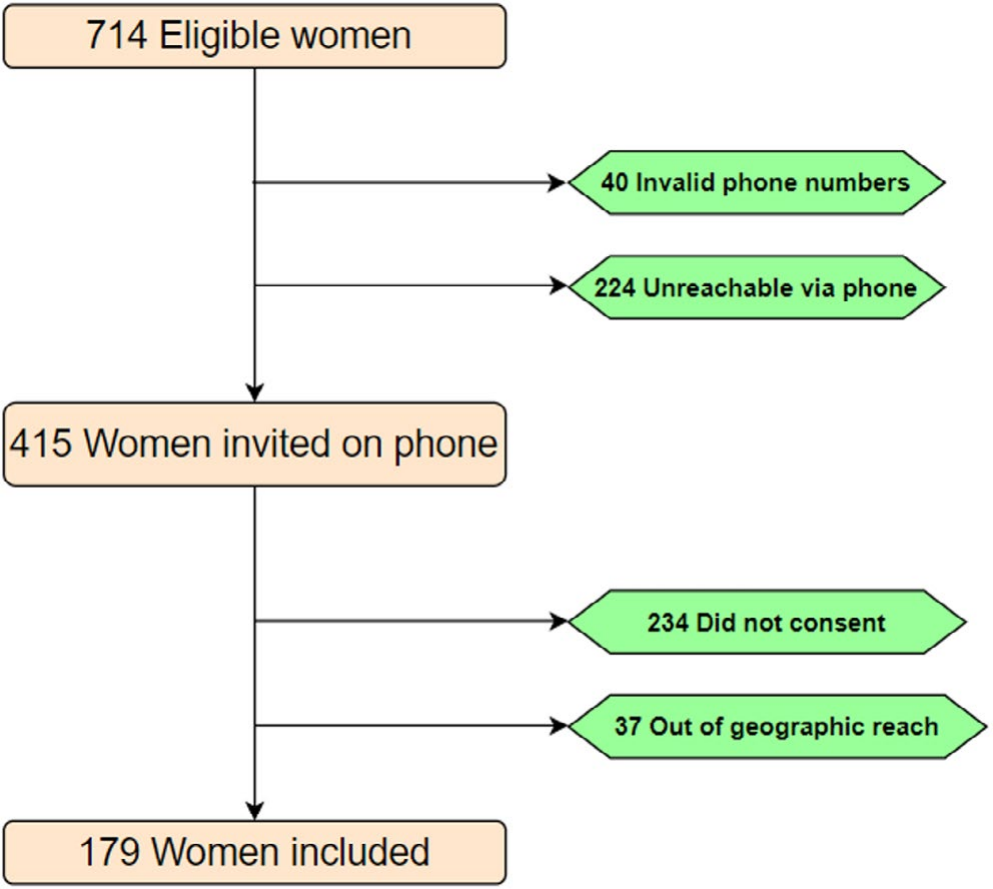
Inclusion flow diagram of women who received family planning care in the public health facilities of the Mifi Health District (West Region of Cameroon); August 2024–November 2024 (*N* = 179).

Of the 714 women we attempted to contact, 40 were registered with invalid telephone numbers and 224 were not reachable despite numerous attempts; Out of the 413 reached by telephone, 37 were geographically out of reach while 234 did not agree to meet the enumerators citing reasons mainly in line with the insecurity spillovers of situation in the neighboring region where a war has been raging for the past 10 years. Finally, 179 women accepted the appointment and were interviewed face‐to‐face (Figure 1; Table S1). Hence the response rate was 43.3%.

The mean (SD) age of respondents was 33.78 (7.7) years and 86.6% of them (155/179) were in union. A total of 96.7% (173/179) were Christians and 63.1% (113/179) had attended secondary school. The self‐declared monthly income of 42.5% (76/179) respondents was below 100 USD. The median (range) number of children per respondent was 4 (0–10).

Table 1 depicts the characteristics of respondents. For 176 of the 179 (98.3%) respondents, the FP provider during the last visit was a woman. During that last FP visit, 90 (50.3%) women adopted a FP method as follows: subcutaneous progestins implants (60 [66.7%]), intramuscular progestins (26 [28.9%]) and intrauterine cooper device (4 [4.4%]).

**TABLE 1:** Respondents' characteristics. Women who received family planning care in the public health facilities of the Mifi Health District (West Region of Cameroon); August 2024–November 2024 (*N* = 179).

Variables	*n* (%)	*P* values
Marital status		
In union	155 (86.6)	<0.001
Single	20 (11.2)	
Widow	4 (2.2)	
Religion		
Christianism	173 (96.7)	<0.001
Islam	2 (1.1)	
African traditional religions	4 (2.2)	
Level of education		
None	3 (1.6)	<0.001
Primary	25 (97.1)	
Secondary	113 (63.1)	
Tertiary	38 (21.2)	
Occupation		
Jobless	52 (29.1)	<0.001
Student	10 (5.6)	
Civil servant	22 (12.3)	
Corporate worker	25 (13.9)	
Self‐employed	70 (39.1)	
Monthly income (USD)		
0	80 (44.7)	<0.001
1–100	76 (42.5)	
101–300	13 (7.3)	
301–500	8 (4.5)	
>500	2 (1.1)	
Categories of health facilities		
First level primary health facilities (IHCs)	28 (15.6)	<0.001
Second level primary health facilities (SMCs)	66 (36.9)	
First level referral (MDH)	31 (17.3)	
Second level referral (BRH)	54 (30.2)	

Abbreviations: BRH, Bafoussam Regional Hospital; IHCs, integrated health centers; MDH, Mifi District Hospital; SMCs, subdivisional medical centers; USD, US dollars.

### 3.2 Woman‐centeredness of FP services ratings

Table 2 summarizes respondents' answers to the survey questions.

**TABLE 2:** Women's ratings of the woman‐centeredness of family planning services in the Mifi Health District (West Region of Cameroon) August 2024–November 2024 (*N* = 179).

Variables	*n* (%)	*P* values
*Autonomy and respect*		
FP provider's self‐introduction		<0.001
No	49 (27.4)	
Yes	130 (72.6)	
FP provider calling women by preferred names		<0.001
No	82 (45.8)	
Yes	97 (54.2)	
Appraisal of waiting time to receive FP care		
Very short	83 (46.3)	<0.001
Somewhat short	66 (36.9)	
Somewhat long	22 (12.3)	
Very long	8 (4.5)	
Feeling of being treated respectfully by the FP provider		
No	5 (2.78)	<0.001
Yes	174 (97.2)	
A few times	4 (2.3)	<0.001
Most of the time	3 (1.7)	
All the time	167 (96.0)	
Feeling of being treated friendly by the FP provider		
No	15 (8.4)	<0.001
Yes	164 (91.6)	
A few times	6 (3.6)	<0.001
Most of the time	7 (4.2)	
All the time	151 (92.2)	
Feeling that the FP provider cared about women		
No	12 (6.7)	<0.001
Yes	167 (93.3)	
A few times	7 (4.1)	<0.001
Most of the time	4 (2.4)	
All the time	156 (93.5)	
Feeling that personal information with be kept confidential by the FP provider		
No	5 (2.8)	<0.001
Yes	174 (97.2)	
A few times	3 (1.7)	<0.001
Most of the time	3 (1.7)	
All the time	168 (96.6)	
Feeling of being given enough information on their care by the FP provider		
No	26 (14.5)	<0.001
Yes	153 (85.5)	
A few times	7 (4.5)	<0.001
Most of the time	14 (9.0)	
All the time	132 (86.5)	
Feeling of understanding the purpose of tests and medicines		
No	33 (18.4)	<0.001
Yes	146 (81.6)	
A few times	9 (6.2)	<0.001
Most of the time	12 (8.2)	
All the time	125 (85.6)	
Feeling of being involved in the choice of FP method		
No	3 (1.7)	<0.001
Yes	176 (98.3)	
A few times	2 (1.1)	<0.001
Most of the time	5 (2.8)	
All the time	169 (96.1)	
Feeling of being given clear explanations by FP providers		
No	27 (15.1)	<0.001
Yes	152 (84.9)	
Most of the time	7 (4.6)	<0.001
All the time	145 (95.4)	
Feeling that the FP provider used intelligible language		
No	12 (6.7)	<0.001
Yes	167 (93.3)	
A few times	1 (0.6)	<0.001
Most of the time	6 (3.6)	
All the time	160 (95.8)	
Feeling that FP providers discussed of women's feelings		
No	16 (8.9)	<0.001
Yes	163 (91.1)	
A few times	3 (1.8)	<0.001
Most of the time	3 (1.8)	
All the time	157 (96.2)	
Feeling that the FP provider gave support for women's fears and anxieties		
No	26 (14.5)	<0.001
Yes	153 (85.5)	
A few times	3 (2.0)	<0.001
Most of the time	2 (1.3)	
All the time	148 (96.7)	
Feeling comfortable to ask the FP provider any question		
No	10 (5.6)	<0.001
Yes	169 (94.4)	
Most of the time	1 (0.6)	<0.001
All the time	168 (99.4)	
Women allowed to have a relative in the FP consultation room		
No	s	<0.001
Yes	98 (54.7)	
A few times	10 (10.2)	<0.001
Most of the time	3 (3.1)	
All the time	85 (86.7)	
Women's being asked about pain by the FP provider		
No	8 (4.5)	<0.001
Not applicable	162 (90.5)	
Yes	9 (5.0)	
A few times	3 (33.3)	<0.001
Most of the time	1 (11.1)	
All the time	5 (55.6)	
Provision of painkillers to women on request		
No	7 (3.9)	<0.001
Not applicable	163 (91.0)	
Yes	9 (5.0)	
A few times	1 (11.11)	<0.001
Most of the time	3 (33.33)	
All the time	5 (55.56)	
Feeling that providers paid attention to them during their stay at the facility		
No	10 (5.6)	<0.001
Yes	169 (94.4)	
A few times	1 (0.6)	<0.001
Most of the time	7 (4.1)	
All the time	161 (95.3)	
The FP provider asked the woman for bribe		
No	172 (96.1)	<0.001
Yes	7 (3.9)	
Woman feeling that the health facility was sufficiently staffed?		
No	29 (16.3)	<0.001
Yes	149 (83.7)	
Woman feeling that health care providers took best care of her?		
No	5 (2.8)	<0.001
Yes	174 (97.2)	
A few times	1 (0.6)	<0.001
Most of the time	6 (3.4)	
All the time	167 (95.9)	
Woman feeling that health care providers cared about her as a person		
No	10 (5.6)	<0.001
Yes	169 (94.46)	
A few times	2 (1.2)	<0.001
Most of the time	5 (2.9)	
All the time	162 (95.9)	
Woman completely trusting health care providers		
No	12 (6.7)	<0.001
Yes	167 (93.3)	
A few times	3 (1.8)	<0.001
Most of the time	4 (2.4)	
All the time	160 (95.8)	
*Health facility environment*		
Overall cleanliness		
Very dirty	1 (0.6)	<0.001
Dirty	8 (4.5)	
Clean	152 (84.9)	
Very clean	18 (10.0)	
Cleanliness of health facility toilets and washrooms		
No	12 (6.7)	<0.001
Yes	58 (32.4)	
Does not know	109 (60.9)	
Presence of water in the health facility		
No	8 (4.5)	<0.001
Does not know	62 (34.6)	
Yes	109 (60.9)	
Presence of electricity in the health facility		
No	1 (0.6)	<0.001
Does not know	9 (5.0)	
Yes	169 (94.4)	
Women feeling safe in the health facility		
No	5 (2.8)	<0.001
Yes	174 (97.2)	
A few times	7 (4.0)	<0.001
Most of the time	7 (4.0)	
All the time	160 (92.0)	
Feeling that the counseling room was overcrowded		
No	131 (73.2)	<0.001
Yes	48 (26.8)	
A few times	29 (60.4)	<0.001
Most of the time	6 (12.5)	
All the time	13 (27.1)	

Abbreviation: FP, family planning.

Table 3 depicts the woman‐centeredness scores of FP care for the whole health district disaggregated by category of health facilities. The median score (range) for the whole district for the full scale was 73.33% (12.22–88.88) while it stood at 61.11% (5.55–100) and 76.38% (8.33–91.66) for facility environment and for women's autonomy and respect respectively. Besides, median scores for all levels of health facilities were lower for the environment than for women's autonomy and respect. The first‐level primary health care facilities (integrated health centers [IHCs]) had lower median scores than other facilities for women's autonomy and respect. Regarding the environment of the facilities, the median score of the first level referral hospital (MDH), was significantly higher than first‐level primary health care facilities.

**TABLE 3:** Women's ratings of the woman‐centeredness of family planning services by categories of health facilities in the Mifi Health District (West Region of Cameroon) August 2024–November 2024 (*N* = 179).

Health facilities	Median scores (ranges)
Autonomy and respect scale (0–100)	
Whole health district	76.38 (8.33–91.66)
First level primary health facilities (IHC)	70.83 (41.66–84.72)
Second level primary health facilities (SMC)	79.16 (8.33–91.66)
First level referral hospital (MDH)	79.16 (68.05–87.5)
Second level referral hospital (BRH)	79.16 (13.88–88.88)
Health facility environment scale (0–100)	
Whole health district	61.11 (5.55–100)
First level primary health facilities (IHC)	61.11 (33.33–83.33)
Second level primary health facilities (SMC)	61.11 (27.77–94.44)
First level referral hospital (MDH)	72.22 (27.77–100)
Second level referral hospital (BRH)	61.11 (5.55–83.33)
Full women‐centered scale (0–100)	
Whole health district	73.33 (12.22–88.88)
First level primary health facilities (IHC)	67.22 (45.55–84.44)
Second level primary health facilities (SMC)	75.55 (15.55–88.88)
First level referral hospital (MDH)	75.55 (66.66–85.55)
Second level referral hospital (BRH)	70.0 (14.22–84.44)

Abbreviations: BRH, Bafoussam Regional Hospital; IHCs, integrated health centers; MDH, Mifi District Hospital; SMCs, subdivisional medical centers.

The calculated cutoff value of woman‐centeredness score interpretation was 50.56% and scores equal to or higher than that cutoff were considered high while those below the cutoff were labeled as low. We found that 93.85% of all respondents had high scores indicating that FP services in the MHD were quite centered on women's needs and values.

### 3.3 Bivariate findings

Table 4 illustrates the results of bivariate linear regression carried out to assess associations between the woman‐centeredness of FP care and several health system and woman‐related characteristics. Only the level of the health facility was found significantly associated as follows: the women‐centeredness score of FP care at the first level referral hospital (MDH) was eight‐point higher than in first level primary care health facilities (crude regression coefficient [cCoef.] = 8.22; 95% CI: 2.46 to 13.93; *P* = 0.005). The women‐centeredness score of FP care in the second level primary health care facilities (subdivisional medical centers) was close to significantly five‐points higher than in first level primary care health facilities (cCoef. = 4.94; 95% CI: −0.04 to 9.91; *P* = 0.051). The women‐centeredness score of FP care at the second level referral health facility was not different from that at the first level primary facilities (cCoef. = −1.3; 95% CI: −6.42 to 3.84).

**TABLE 4:** Correlates of the woman‐centeredness of family planning services from bivariate linear regression models in the Mifi Health District (West Region of Cameroon) August 2024–November 2024 (*N* = 179).

Variables	Crude regression coefficients (95% CI)	*r* ^2^	*P* value
Health facility			
First level primary health facilities (IHC)	Ref.	0.1	Ref.
Second level primary health facilities (SMC)	4.94 [−0.036; 9.91]		0.051
First level referral hospital (MDH)	8.22 [2.46; 13.93]		0.005
Second level referral hospital (BRH)	−1.3 [−6.42; 3.84]		0.61
Level of education			
Tertiary	Ref.		Ref.
None	−11.53 [−25.24; 2.18]		0.09
Primary	2.23 [−3.65; 8.12]		0.45
Secondary	3.32 [−1.96; 6.61]		0.28
Marital status			
In union	Ref.		Ref.
Single	−1.15 [−6.62; 4.32]		0.67
Widow	5.96 [−5.71; 17.63]		0.31
Occupation			
Jobless	−2.54 [−6.77; 1.68]	0.01	0.23
Corporate worker	−3.91 [−9.29; 1.47]		0.15
Civil servant	−1.87 [−7.52; 3.76]		0.51
Student	−1.88 [−9.69; 5.91]		0.63
Monthly income (USD)			
1–100	−1.21 [−4.92; 2.50]	0.01	0.52
101–300	−2.91 [−9.84; 4.02]		0.4
301–500	1.36 [−7.23; 9.95]		0.75
Gestity	−0.49 [−1.24; 0.25]	0.01	0.19
Parity	−0.34 [−1.16; 0.47]	0.01	0.4
Family planning provider's gender			
Male	−0.53 [−13.96; 12.89]	0.01	0.93
Selected family planning method			
None	Ref.	0.04	Ref.
Intrauterine copper device	−5.91 [−17.63; 5.72]		0.31
Subcutaneous progestins implant	2.58 [−1.25; 6.41]		0.18
Intramuscular progestins	−1.91 [−7.02; 3.19]		0.45

Abbreviations: BRH, Bafoussam Regional Hospital; CI, confidence interval; IHC, integrated health centers; MDH, Mifi District Hospital; Ref, reference category; SMC, subdivisional medical centers; USD, US dollars.

### 3.4 Multivariate findings

On multivariate linear regression whose results are depicted in Table 5, the woman‐centeredness of FP care at the first‐level referral hospital (MDH) was found to be significantly seven‐point higher than in first level primary health care facilities (adjusted regression coefficient [aCoef.] = 7.33; 95% CI: 1.11 to 13.56; *P* = 0.02). Conversely, the woman‐centeredness of FP care was significantly seven‐point lower for jobless women (aCoef. = −7.84; 95% CI: −14.14 to −1.55; *P* = 0.01) and for women earning ≤100 USD/month (aCoef: –6.98; 95% CI: −12.96 to −1.01; *P* = 0.02) than for those earning >300 USD/month. None of the other exposure variables was associated with a significant change in the woman‐centeredness score.

**TABLE 5:** Correlates of the woman‐centeredness of family planning services from multivariate linear regression models in the Mifi Health District (West Region of Cameroon) August 2024–November 2024 (*N* = 179).

Variables	Adjusted odds ratio (95% CI)	*P* value
Health facility (first level referral hospital [MDH])	7.33 [1.11; 13.56]	0.02
Occupation (civil servant)	1.34 [−5.89; 8.59]	0.71
Occupation (jobless)	−7.84 [−14.14; −1.55]	0.01
Monthly income (0–100 USD)	−6.98 [−12.96; −1.01]	0.02
Monthly income (101–300 USD)	−6.50 [−15.39; −2.38]	0.15
Family planning provider gender (male)	1.30 [−13.22; 15.84]	0.85

Abbreviations: CI, confidence interval; MDH, Mifi District Hospital; USD, US dollars.

## 4 DISCUSSION

### 4.1 Main results

Using a comprehensive and integrated scale, this study found a quite high level of woman‐centeredness of FP services in the MHD with significant differences between categories of health facilities. Specifically, ratings related to woman's respect and autonomy were higher than those related to health facility environment. Regarding the correlates of woman‐centeredness, FP care was more centered on women at the district hospital than in first‐level primary health facilities; Likewise, FP care was more centered on women with higher income. Worth noting is the fact that the degree of woman‐centeredness of FP care at the second level referral hospital (BRH) was not different from that at the first‐level primary health facilities.

### 4.2 Results in context

The response rate in this study (43.3%) might seem low but is ordinary given its closeness to the 44.1% average reported by Wu et al. in a meta‐analysis of published online surveys.^33^ In fact, recruitment through phone calls used in this study and recruitment via online tools are both indirect approaches that elucidate the same behavioral reactions thereby yielding similar response rates. Moreover, considering the deteriorated security context of the West Region caused by the raging war in two neighboring regions, the observed response rate should be considered high.

The high woman‐centeredness nature of FP services found in this study denotes in the country's health system critiqued by its own Ministry of health for its generalised inadequate quality of services.^34^ Moreover, that result is in sharp contrast with those from several studies in SSA depicting the superiority in quality of FP services in private facilities over state‐owned ones.^26^, ^34^ The commonest picture in SSA is quite different, with FP services displaying little woman‐centeredness illustrated by a Ugandan series reporting that more than two‐thirds of women were neither willing to return to their FP care providers nor to recommend them to a relative or friend.^25^, ^27^, ^35^, ^36^ The reason why this study revealed such a contrasting albeit data‐grounded degree of woman‐centeredness probably lies in the comprehensiveness of the measurement scale which operated a more itemized, integrated and balanced appraisal of such a complex construct. Indeed, it has been established that individuals do not spontaneously perceive all the components of constructs like people‐centeredness at the expense of the more objective elements.^37^ This results in an increased likelihood of unbalanced assessment when measurement tools are not robust. We believe that the use of that validated scale in this study integrated the objective components in the model thereby redressing the poor picture women frequently depict when judging FP and health care services at large. It is worth emphasizing that the said scale encompasses most items of the structure and service‐giving processes set out in international quality standards for FP care.^17^, ^18^, ^19^, ^20^ In support of that explanation, a study conducted in Ethiopia using a similar scale also found a high quality of FP services supported by strong woman‐centeredness elements.^38^ Could the use of validated measurement scales be a game changer in appraising the quality of health services in Cameroon?

The study found that ratings of women's autonomy and respect were significantly higher than those related to health facility physical environment. That deplorable finding regarding infrastructural features of facilities is in line with the Cameroon's health system characteristics.^39^ Women's respect and autonomy was understandably rated high considering that more than half of respondents came from primary health care facilities which are known to be staffed with care providers having a sociocultural proximity with users. In support of this reading is the fact that only 4% of respondents reported being asked for a bribe in a national context marred by corruption.^40^, ^41^

FP care was less user‐centered for women with low or no income care, underscoring a well‐known and sensitive inequity: poor quality health care services for poor people. Several facets of that injustice have been reported in Cameroon in other segments of sexual and reproductive health and beyond: access to antenatal care, sustainability of cesarean section expenditures, quality of postnatal care, and affordability of universal health coverage.^42^, ^43^, ^44^, ^45^ Additional scrutiny of that issue is necessary to gather more robust conclusions to inform action in a region that has taken many internally displaced people form the neighboring conflict‐torn regions.^46^

FP services at the MDH which is the first level referral hospital of the district were more women‐centered than in other health facilities. In this regard, the MDH preaches by example; In fact, its FP unit is led by a senior sexual and reproductive nurse who serves as a regional tutor for quality improvements in FP services. In that capacity, the unit serves as a center for practical in‐service training of FP providers from across the West Region.^47^

Conversely, the level women‐centeredness of FP at the BRH (the top referral in the West Region) was not different from that in the first‐level primary health facilities. While this seems counter‐intuitive, several factors from the list reported by Tessema et al. in their systematic review of the determinants of quality of FP services in Africa could provide valid explanations.^1^ This was not within the scope of this study.

### 4.3 Implications for policy and practice

The highly women‐centered nature of FP care revealed by this study is a testament to good practices in care delivery in public health facilities in the MHD. In a learning health system endeavor, policy makers, planers and implementers should map out the underlying mechanisms of such a positive outcome and scale them across the country.^48^ In addition, within the MHD, FP care providers should pay special attention to users with low socioeconomic background.

### 4.4 Strengths

This study exhibits several strengths worth noting. First, to the best of author's knowledge, it stands as the pioneer in assessing FP services from a user's perspectives in the West Region of Cameroon. Second, the scale used has been developed in a setting with similar socio‐cultural and economical features.^30^ Third, proportional sampling ensured a good representativity of FP users in the catchment areas of all the public health facilities of the MHD. Fourth, the social the desirability bias was minimized by conducting data collection outside health facilities by purposefully selected and trained non‐health professionals enumerators using a forced‐choice items scale.^49^, ^50^

### 4.5 Limitations

The most significant limitations to this study include a substantial risk of selection bias and a single‐faceted appraisal of the quality of FP services. Regarding the selection bias, the moderate response rate and the non‐probability selection of women within health facilities limit generalizability of findings; Furthermore, one third of eligible women were not reachable through phone which was the unique channel for recruitment. This could have excluded FP users of lower socioeconomic status, who are less likely to own phones and more likely to live in remote areas with low phone network coverage. The potential distortion of results by that bias is an overestimation of the observed woman‐centeredness scores given that women of low‐income background were more likely to provide low ratings. With respect to the single‐faceted approach, this study mainly assessed the outcome of service delivery while a holistic evaluation would have encapsulated structure indicators and provided a broader picture of the quality of FP services.^18^

### 4.6 Future research directions

Moving forward, qualitative research is needed to explore the lower quality of FP services offered to women of low socioeconomic background. Likewise, it is worthy to investigate why the highest‐level health facility does not deliver the highest level of woman‐centeredness of FP care. Finally, it is tantamount to elucidate the mechanisms underpinning the high woman‐centeredness of FP services in the MDH.

## 5 CONCLUSION

This study used a comprehensive and integrated survey scale and found that FP services in public health facilities were highly centered on women's needs and values. However, FP care in the district hospital was more women‐centered than in primary care health facilities probably due the presence of a FP champion. This study also revealed that FP care was less centered on women with lower income, hence raising an issue of equity.

## AUTHOR CONTRIBUTIONS

JTF, WCDK and ATT: Conceptualization, data acquisition, analysis and interpretation. JTF, WCDK, ATT, YFF, MAND, EDMK and JHFF: Drafting the manuscript oand reviewing it critically for important intellectual content and final approval of the version of the manuscript to be published. JTF and JHFF: Agree to be accountable for all aspects of the work.

## FUNDING INFORMATION

This research was funded with support from the Center for International Reproductive Health Training at University of Michigan (CIRHT‐UM). Pre‐Publication Support Service (PREPSS) supported the development of this manuscript.

## CONFLICT OF INTEREST STATEMENT

The authors have no conflicts of interest.

## Supporting information


**Table S1.** Distribution of respondents by health facilities.

## Data Availability

Research data are not shared.
