# Supplementary material for: Woman‐centeredness of family planning care and associated factors in a semi‐urban health district in West Cameroon
Source: Int J Gynaecol Obstet. 2025 Nov 17;173(2):763–73. doi: 10.1002/ijgo.70654 (PMC13094692; doi:10.1002/ijgo.70654)
Supplement: Supplementary file 1 — Table S1. Distribution of respondents by health facilities. [file IJGO-173-763-s001.docx]

Supplementary material

Table S1: Distribution of respondents by health facilities

| **Health facilities** | New FP users | Registered new FP users | Registered new FP users called on phone | New FP users registered under wrong phone numbers | Not available on phone | Women not available | Refusal to participate | Geographically out of reach | Included in the survey |
| --- | --- | --- | --- | --- | --- | --- | --- | --- | --- |
| **Badiembou IHC** | 14. | 10 | 10 | 0 | 0 | 2 | 5 | 0 | 3 |
| **Banefo IHC** | 4. | 13 | 13 | 0 | 0 | 10 | 0 | 0 | 3 |
| **Batoukop IHC** | 11. | 16 | 16 | 1 | 1 | 0 | 3 | 1 | 6 |
| **Djeleng SMC** | 38. | 100 | 90 | 1 | 22 | 10 | 40 | 2 | 15 |
| **Mifi DH** | 250. | 100 | 100 | 7 | 55 | 18 | 6 | 9 | 32 |
| **Keuleu IHC** | 19. | 11 | 11 | 1 | 0 | 2 | 5 | 2 | 1 |
| **Banengo Village IHC** | 9. | 13 | 13 | 0 | 0 | 2 | 6 | 0 | 2 |
| **King place IHC** | 32. | 30 | 20 | 1 | 5 | 0 | 4 | 0 | 10 |
| **Kongso SMC** | 18. | 10 | 10 | 0 | 0 | 0 | 7 | 0 | 3 |
| **Lafe-Baleng SMC** | 240. | 110 | 100 | 4 | 57 | 3 | 6 | 2 | 32 |
| **Bafoussam RH** |  | 210 | 205 | 11 | 85 | 30 | 8 | 18 | 53 |
| **Tyo ville SMC** | 83. | 110 | 110 | 13 | 28 | 55 | 7 | 6 | 14 |
| **Yagou IHC** | 22. | 9 | 9 | 1 | 0 | 0 | 1 | 0 | 5 |
| **Total** | 753 | 749 | 714 | 40 | 259 | 133 | 98 | 4 | 179 |

IHC:Integrated health center; SMC: subdivisional medical center; RH:Regional hospital; DH: District hospital; FP: Family planning
